# Healthcare Costs and Mortality Trends of Elderly ICU Patients: Evidence from an Eight-Year Cohort Study in China

**DOI:** 10.3390/healthcare14030364

**Published:** 2026-01-31

**Authors:** Xiaohui Zhu, Meiping Wang, Yawei Guo, Li Jiang

**Affiliations:** 1 Emergency Department, Xuanwu Hospital of Capital Medical University, Beijing 100053, China; 2 Department of Critical Care Medicine, Xuanwu Hospital of Capital Medical University, Beijing 100053, China

**Keywords:** elderly patients, intensive care unit, healthcare costs, surgical status, mortality, temporal trends

## Abstract

**Background/Objectives:** Elderly patients represent a growing proportion of ICU admissions, raising concerns about outcomes and healthcare costs. Evidence from China remains limited, yet understanding cost and mortality patterns is critical for optimizing care in aging populations. **Methods:** We retrospectively analyzed 31,535 ICU patients admitted from 2014 to 2021. After 1:1:1 matching on severity, comorbidities, sex, and admission type, three groups were formed: elderly (≥80 years), older (65–79 years), and younger (16–64 years), with 3398 patients each. Costs were inflation-adjusted, and outcomes compared across groups with appropriate statistical models. **Results:** Elderly patients accounted for 11.5% of admissions, had the longest ICU stay (4.5 vs. 3.8 vs. 3.1 days, *p* < 0.001), and the highest ICU (11.5%) and hospital (13.5%) mortality. Among non-surgical patients, elderly incurred the lowest costs; however, surgery reversed this pattern, producing a 124% increase. Expenditures were mainly driven by drugs and consumables. From 2014 to 2021, consumables rose from 32.0% to 42.0% of total costs, whereas drug costs declined. Inflation-adjusted hospital costs remained stable over time, while mortality among elderly patients decreased significantly (19.5% in 2014 vs. 8.8% in 2021; OR 0.86 per year, *p* < 0.001). **Conclusions:** Elderly ICU patients demonstrate unique cost and outcome profiles. While non-surgical elderly patients are less costly, surgery substantially increases expenses. Mortality declined over time without a rise in real costs, suggesting improved efficiency of critical care. These findings support tailored resource allocation and policy planning for aging ICU populations.

## 1. Introduction

The intensive care unit (ICU) is one of the most resource-intensive hospital departments, accounting for nearly 20–30% of all inpatient costs [1]. Daily ICU costs are roughly three times those of general wards [2]. With rapid population aging, patients aged ≥80 years now represent an increasingly large proportion of ICU admissions worldwide, including in China [3,4,5,6].

Elderly ICU patients often experience worse clinical outcomes than younger patients, including higher mortality and poorer functional recovery [3,7,8]. These concerns have intensified ethical and policy debates over the cost-effectiveness and appropriateness of intensive care in elderly patients [9,10]. Over the past decade, numerous studies have investigated treatment intensity, prognostic factors, and outcomes of elderly ICU patients [11]. However, evidence on healthcare costs remains limited, particularly in comparisons with younger, severity-matched cohorts. Moreover, prior studies have reported inconsistent findings on whether advanced age is associated with higher or lower healthcare expenditures [12,13]. Clarifying the financial burden of ICU care for elderly patients is therefore essential for informing resource allocation and shaping critical care policy in aging societies.

In parallel, healthcare delivery in China has changed substantially in recent years. Advances in surgical techniques, therapeutics, and supportive care, along with reforms in medical insurance and hospital management, may have altered both ICU expenditure patterns and patient outcomes. However, how these system-level developments have affected elderly ICU patients remains unclear, particularly because resource utilization may differ markedly across age groups.

Accordingly, we conducted a retrospective cohort study using data from a tertiary teaching hospital in Beijing to evaluate healthcare costs and outcomes among elderly ICU patients relative to younger matched patients. Specifically, we aimed to: (1) examine the association between age group and total hospital costs and assess whether this association is modified by surgical status (i.e., effect modification); (2) characterize the composition of costs and their temporal trends among elderly patients; and (3) evaluate mortality trends over an eight-year period. By quantifying age-related cost patterns within the evolving Chinese healthcare context, this study provides evidence to support clinical decision-making, ICU resource allocation, and policy development for aging ICU populations.

## 2. Materials and Methods

### 2.1. Study Design and Population

This retrospective cohort study was conducted at a tertiary teaching hospital in Beijing, China, with 1643 inpatient beds, including 128 ICU beds. We extracted electronic medical record (EMR) data for all ICU admissions between 1 January 2014 and 31 December 2021 (*n* = 38,417). For patients with multiple ICU admissions (*n* = 5863), only the first admission was included. We excluded patients aged < 16 years (*n* = 1011) and those with missing cost data or key matching variables (*n* = 8). The final unmatched cohort included 31,535 patients.

Patients were classified into three age groups at ICU admission: elderly (≥80 years; *n* = 3624), older (65–79 years; *n* = 9679), and younger (16–64 years; *n* = 18,232). All eligible patients aged ≥ 80 years were included in the elderly group. Elderly patients were subsequently matched 1:1:1 to patients aged 65–79 years and 16–64 years. Matching variables included sex, admission type (surgical vs. medical), Charlson Comorbidity Index (CCI) category, and illness severity at admission (Acute Physiology Score [APS] within ±1). When multiple eligible controls were available, one control was randomly selected. After excluding 226 unmatched elderly patients, the final matched cohort included 3398 patients in each age group (total *n* = 10,194).

### 2.2. Variables

Illness severity at ICU admission was assessed using the Acute Physiology Score (APS), calculated by subtracting age and chronic health/admission-type points from the Acute Physiology and Chronic Health Evaluation II (APACHE II) score. Comorbidities were identified from pre-existing diagnoses based on the 19 conditions included in the Charlson Comorbidity Index (CCI), using hospital discharge codes recorded in the medical records database. The CCI was categorized as low (0), moderate (1–2), or high (≥3). Functional status at ICU admission was assessed using the Activities of Daily Living (ADL) score, with a range from 0 (complete dependence) to 100 (complete independence), based on nursing evaluation within 24 h of admission.

Primary diagnoses were grouped into eight categories: cardiovascular, respiratory, gastrointestinal, neurologic, infectious, musculoskeletal/injury-related, hematologic/oncologic, and other diseases [14]. Organ support was defined as the use of invasive mechanical ventilation, renal replacement therapy (RRT), inotropics/vasopressors (epinephrine, norepinephrine, dopamine, metaraminol, and dobutamine), nutrition support, and blood transfusion during ICU stay. We also included ICU and hospital length of stay, ICU and hospital mortality, and insurance status (insured vs. self-financed). Cost data were obtained from the electronic billing database, including total hospitalization costs and itemized expenditures. Expenditures were categorized into eight components: drugs; surgery; laboratory tests; imaging; consumables; bed, monitoring, and oxygen; nursing care; and other costs. To account for inflation during the study period, costs were adjusted using the Beijing Consumer Price Index (CPI) and reported in constant 2014 Chinese yuan (CNY). Annual trends were estimated based on year of ICU admission. For patients whose hospitalization spanned multiple calendar years, all costs were assigned to the year of first ICU admission. All variables required for analysis were complete.

### 2.3. Statistical Analysis

Continuous variables are presented as mean (SD) or median (IQR), and categorical variables as frequencies (percentages). Due to right-skewed distribution, healthcare costs were natural log-transformed for inferential analyses. Analyses were performed using SPSS version 25.0 and STATA 18.0 (Stata, College Station, TX, USA), with a two-sided *p* < 0.05 considered significant.

Balance across matching variables (sex, admission type, CCI category, and APS) was assessed using standardized mean differences (SMDs), with SMD < 0.10 indicating adequate balance. Characteristics of unmatched elderly patients (*n* = 226) were compared with those of matched elderly patients to evaluate potential selection bias. For descriptive comparisons across matched age groups, log-transformed costs were analyzed using repeated-measures analysis of variance (RM-ANOVA). Although formal normality tests on residuals were statistically significant (Shapiro–Wilk *p* < 0.001), visual inspection of normal P–P plots indicated acceptable approximation to normality for descriptive purposes. The sphericity assumption was met (Mauchly’s test: W = 1.000, *p* = 0.581). Non-normally distributed continuous variables (e.g., length of stay) were compared using Friedman tests, followed by Bonferroni-adjusted Wilcoxon signed-rank tests for pairwise comparisons. Categorical variables were compared using Cochran’s Q test, with Bonferroni-adjusted McNemar tests for post hoc comparisons. Within the elderly group, costs were compared using independent-samples *t*-tests after log transformation.

To assess whether surgical status modified the association between age group and total costs, we fitted a linear mixed-effects model accounting for matched sets (pair ID as a random intercept). The model was adjusted for primary diagnosis category and included an age group × surgical status interaction term. Model fit was evaluated via likelihood ratio test (LRT), intraclass correlation coefficient (ICC), and R^2^. Regression coefficients (β) represent adjusted mean differences in log-costs and were back-transformed to percentage changes using [exp(β) − 1] × 100%. The interaction effect is visually represented in the marginal means plot. Simple effects analysis was conducted if the interaction was significant.

Model assumptions were assessed using standard residual diagnostics. Although normal P–P plots suggested approximate normality, heteroscedasticity was detected based on residual plots and the Breusch–Pagan test (*p* < 0.001). Therefore, all inferences were based on heteroscedasticity-robust (Huber–White) standard errors. To evaluate robustness, sensitivity analyses were performed using the full unmatched cohort (*n* = 31,535) with multivariable linear regression adjusting for age group, surgical status, their interaction, sex, CCI category, APS, and primary diagnosis. Additional sensitivity analyses included excluding influential observations (Cook’s distance > 4/N), nonparametric bootstrapping (500 replications), and further adjustment for baseline functional status (ADL score).

Temporal trends in inflation-adjusted (real) costs among elderly patients were assessed using multivariable linear regression with year of admission as a continuous predictor, adjusted for age, sex, CCI category, APS, surgical status, and primary diagnosis. To separate the effects of price inflation from changes in resource utilization, the same model was applied to both CPI-adjusted (real) and unadjusted (nominal) costs. Temporal trends in in-hospital mortality among elderly patients were examined using multivariable logistic regression with the same covariates, and further adjustment for ADL. Cost composition trends were presented graphically.

### 2.4. Ethics

The study was approved by the Human Research Ethics Committee of Xuanwu Hospital, Beijing, China (approval number: 2024(047)-001). Given the retrospective design and use of de-identified data, the requirement for informed consent was waived. Data were accessed from the hospital EMR system between 1 May 2024 and 8 October 2024. Ethical approval was valid from 28 April 2024 to 28 April 2025. Investigators were unable to identify individual participants during or after data collection.

## 3. Results

**Figure 1:**
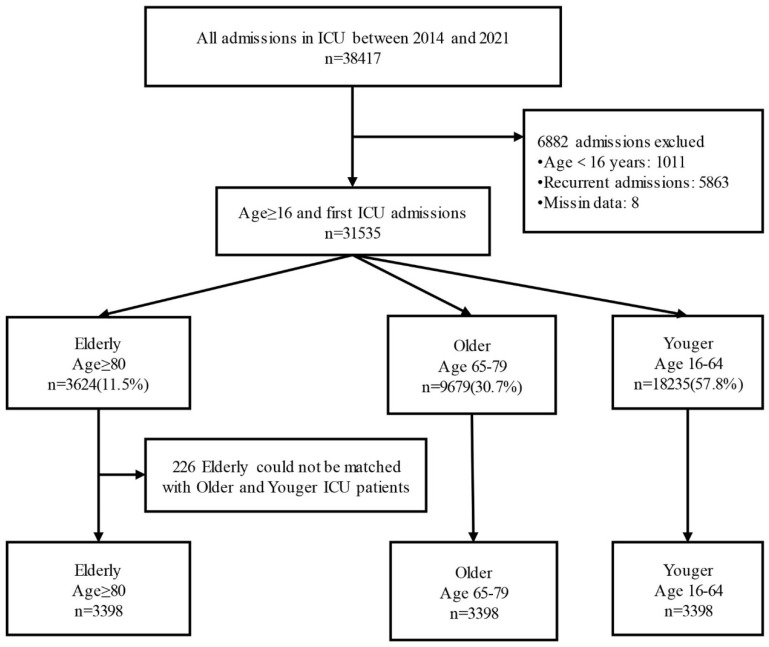
Study diagram.

We identified 31,535 first ICU admissions among patients aged ≥16 years at Xuanwu Hospital, Beijing, between 1 January 2014 and 31 December 2021. Of these, 3624 were elderly patients, accounting for 11.5% of all ICU admissions. After 1:1:1 matching, three study groups were formed, each including 3398 patients, with 226 elderly patients (6.2%) excluded due to lack of eligible matches (Figure 1). Matching performance was excellent, with standardized mean differences (SMDs) <0.01 for all prespecified matching variables (Table S1). Matching distributions are shown in Table 1, and baseline characteristics and ICU course are summarized in Table 2.

Compared with matched older and younger patients, elderly patients were less likely to receive invasive mechanical ventilation or tracheostomy, but more frequently received vasopressors/inotropes, blood transfusion, albumin infusion, and parenteral nutrition (Table 2). Elderly patients had the longest ICU length of stay (median 4.5 vs. 3.8 vs. 3.1 days, *p* <0.001), while hospital length of stay was shorter than that of older patients (median 11.4 vs. 11.9 days, *p* = 0.004). ICU mortality was highest in the elderly group (11.5% vs. 7.2% vs. 4.7%, *p* < 0.001), as was hospital mortality (13.5% vs. 8.3% vs. 5.2%, *p* < 0.001).

### 3.1. Healthcare Costs by Age Group and Surgical Status

Mean total costs and cost components by age group are shown in Table 3. Overall, elderly patients had the lowest mean total and daily healthcare costs compared with matched older and younger patients. However, this pattern differed substantially by admission type. Among medical (non-surgical) patients, elderly patients incurred lower costs than both comparator groups, whereas among surgical patients, costs in elderly patients were comparable to those in older patients and higher than those in younger patients (Table 3).

**Figure 2:**
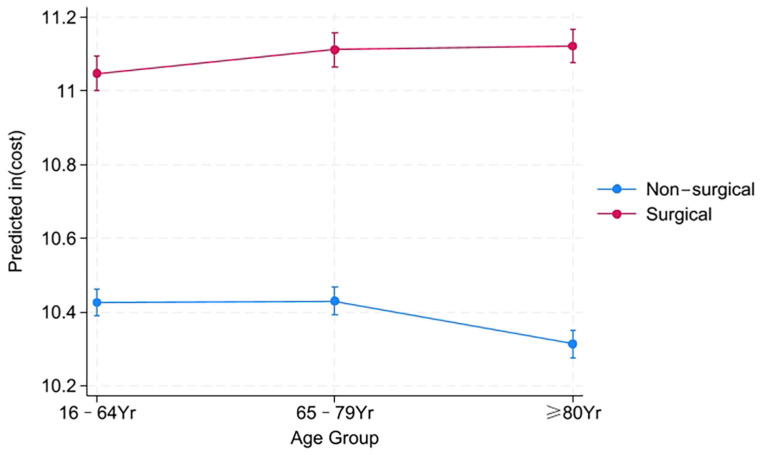
Adjusted marginal means of log-transformed medical costs by age group and surgical status. Notes: Marginal means and 95% confidence intervals were estimated from a linear mixed-effects model accounting for the matched-pair design (with pair ID as a random intercept) and adjusted for primary diagnosis category. The interaction between age group and surgical status was statistically significant (*p* < 0.05). *p*-values for specific pairwise comparisons within surgical strata are presented in Table S3.

To formally assess whether surgical status modified the association between age group and costs, we fitted a linear mixed-effects model accounting for matched sets and adjusted for primary diagnosis (Table S2). A significant age group × surgical status interaction was observed (*p* < 0.001), indicating that the relationship between age and costs depended on the treatment pathway. Among non-surgical patients, elderly patients had significantly lower total costs than younger patients (−13.6%, 95% CI −16.9% to −10.2%; *p* < 0.001) and older patients (−14.4%, 95% CI −17.7% to −11.0%; *p* < 0.001). In contrast, within the elderly cohort, surgical admission was associated with a 124% increase in costs (95% CI 111% to 138%; *p* < 0.001). Consequently, among surgical patients, elderly patients had costs that were 7.1% higher than younger patients (95% CI 0.9% to 13.9%; *p* = 0.023) and did not differ from older patients (−1.0%, 95% CI −7.6% to 5.6%; *p* = 0.762). This interaction is illustrated in Figure 2, with pairwise comparisons presented in Table S3.

Model diagnostics and robustness checks are summarized in Tables S4–S10. The random intercept significantly improved model fit (χ^2^(1) = 26.73, *p* < 0.001), with an intraclass correlation coefficient of 5.3% and a conditional R^2^ of 0.28 (AIC = 25,514, BIC = 25,622). Sensitivity analyses yielded consistent results in the full unmatched cohort (N = 31,535; surgical cost increase: 146%, 95% CI 133% to 160%; *p* < 0.001), after exclusion of influential observations, and using bootstrap validation (500 replications). Inferences were based on heteroscedasticity-robust standard errors (Breusch–Pagan test *p* < 0.001). Additional adjustment for baseline functional status (ADL) did not materially change the results. To assess potential selection bias, unmatched elderly patients (*n* = 226) were compared with matched elderly patients (Table S11). Unmatched patients had greater illness severity and lower surgical rates (0.4% vs. 38.9%), suggesting their exclusion may have introduced a conservative bias.

The financial impact of surgery varied significantly by primary diagnosis (*p* for interaction <0.001). Among patients aged ≥80 years, surgical treatment for cardiovascular disease was associated with a 154% cost increase compared with non-surgical management, whereas the corresponding increase for gastrointestinal disease was 89% (Tables S12 and S13). Among elderly surgical patients, consumables accounted for the largest cost component (56% of total costs), accompanied by longer ICU stays and higher use of vasoactive drugs and blood transfusion compared with younger surgical patients (Table S14).

### 3.2. Cost Predictors and Temporal Trends in Elderly Patients

Subgroup analyses within the elderly cohort are shown in the Supplement (Tables S1–S5). Costs were higher among elderly patients admitted for surgery, in males, and in those with higher CCI scores. Costs did not differ significantly by insurance status (*p* = 0.52) or between survivors and decedents (*p* = 0.18). Multivariable regression within the elderly cohort confirmed these patterns and further showed that costs were lower with increasing age, lower APS, and cardiovascular primary diagnoses (Table 4).

**Figure 3:**
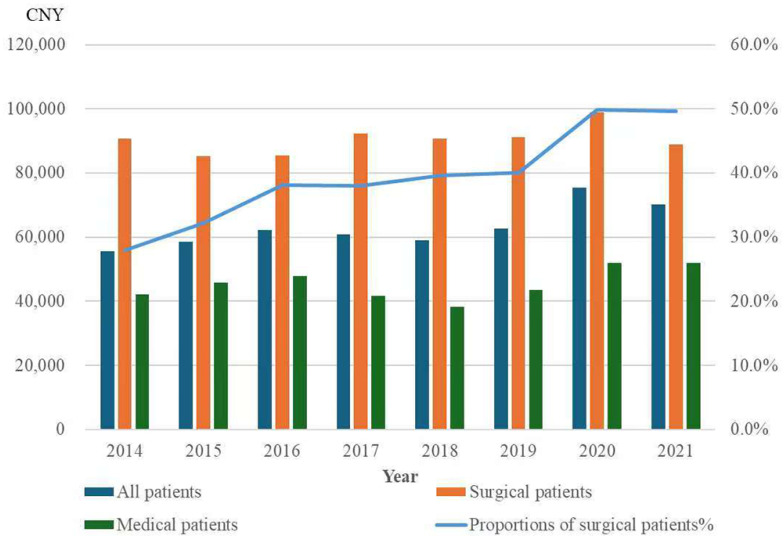
Mean inflation-adjusted (real) healthcare costs for elderly patients and proportion of surgical patients, 2014–2021. The proportion of surgical patients among elderly patients increased annually between 2014 and 2021, accompanied by an overall increase in healthcare costs for the entire elderly patient population (univariate analysis: 95% CI: 0.013 to 0.042, *p* < 0.001). However, subgroup analysis showed that healthcare costs remained stable over time within both surgical and medical patient subgroups.

In unadjusted analyses, inflation-adjusted (real) healthcare costs among elderly patients increased by 2.84% per year (95% CI 0.013 to 0.042; *p* < 0.001), concurrent with an increasing proportion of surgical admissions (Figure 3). However, after adjustment for case-mix factors (age, sex, CCI, APS, surgical status, and primary diagnosis), the temporal trend in real costs was no longer significant (β = −0.001 per year, 95% CI −0.014 to 0.011; *p* = 0.829; Table 4). In sensitivity analyses excluding patients whose hospitalization spanned a year-end (*n* = 144, 4.2%), results were unchanged (β = −0.001; *p* = 0.833; Tables S15 and S16). In contrast, the same adjusted model applied to nominal (unadjusted) costs showed a significant increasing trend (β = 0.018 per year; *p* = 0.006; Tables S17 and S18).

### 3.3. Medical Cost Composition and Its Temporal Evolution in Elderly Patients

**Figure 4:**
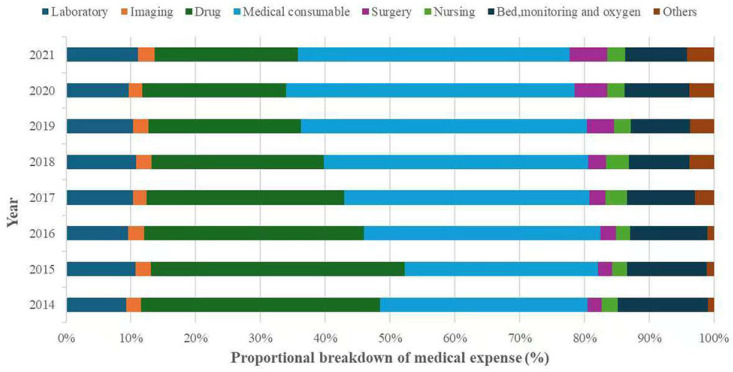
The breakdown of medical expense for elderly ICU patients, 2014–2021. Others include fees for diagnosis, registration and other treatment.

Drugs and consumables were the two largest cost components across all age groups. In elderly patients, drugs accounted for 29.2% and consumables for 38.6% of total costs (Table 3). Over the study period, consumables surpassed drugs as the primary expenditure category. The proportion of consumable costs increased from 32.0% in 2014 to 42.0% in 2021, although the rate of increase slowed after 2019. In contrast, the share of drug costs declined from 36.9% to 22.1%. Surgery fees and other costs increased by 3.6% and 3.2%, respectively, whereas expenditures on laboratory tests, imaging, and nursing care remained relatively stable (Figure 4).

### 3.4. Temporal Trends in Hospital Mortality in Elderly Patients

Unadjusted hospital mortality among elderly patients decreased from 19.5% in 2014 to 8.8% in 2021. In multivariable logistic regression, later year of admission was independently associated with lower odds of in-hospital death (OR 0.86 per year; *p* < 0.001; Table S19), corresponding to a 14% reduction in mortality odds per calendar year. Further adjustment for ADL did not materially change the temporal decline (OR 0.85; *p* < 0.001; Table S20).

## 4. Discussion

In this eight-year retrospective cohort study of ICU patients in a tertiary hospital in Beijing, we found that elderly patients (≥80 years) represented 11.5% of ICU admissions and exhibited distinct patterns of resource use, costs, and outcomes compared with matched older (65–79 years) and younger (16–64 years) patients. Elderly patients had the longest ICU stays but shorter hospital stays than older patients, with significantly higher ICU and in-hospital mortality. Importantly, healthcare costs were lowest in elderly non-surgical patients but increased markedly when surgery was involved, eliminating their relative cost advantage. Over time, inflation-adjusted total costs in elderly ICU patients remained stable, whereas the cost structure shifted considerably, with consumables replacing drugs as the dominant expenditure category. Notably, hospital mortality declined substantially during the study period.

Direct comparisons with previous studies are challenging due to substantial heterogeneity in cost definitions, reporting methods, and healthcare financing systems. As a result, published estimates of ICU costs among elderly patients vary widely [13,15]. Nonetheless, prior studies consistently highlight two key global trends: a growing proportion of elderly patients admitted to ICUs [3], and a considerable economic burden associated with their care [13]. While some studies report that advanced age is associated with lower hospital costs and reduced resource utilization [11,16,17], often alongside higher rates of decisions to limit life-sustaining therapies [15,18,19]. others, such as a Dutch study, note that costs for elderly ICU patients can be higher than Younger ICU patients, or comparable to older ICU patients [13].

Against this complex international backdrop, our findings reveal a nuanced, treatment-dependent relationship between age and costs in the Chinese context. Consistent with reports from several European healthcare systems, elderly non-surgical patients in our cohort incurred the lowest expenditures. However, surgical intervention completely reversed this pattern, increasing costs by 124% among elderly patients. This dramatic escalation aligns with research identifying surgery as a major driver of ICU expenditure, particularly in frail elderly populations [20]. The substantial cost increase associated with surgery in the elderly appears to be driven by a combination of factors: a higher proportional cost of consumables (accounting for 56% of expenses in this subgroup), longer ICU stays, and a higher rate of relatively non-invasive organ support—such as vasoactive drugs and blood products—which likely reflect both procedural complexity and higher baseline vulnerability. Furthermore, the cost premium was not uniform across diagnoses; it was nearly twice as high for cardiovascular disease (154%) as for gastrointestinal disease (89%). This disproportionate effect, combined with the high prevalence of cardiovascular procedures, suggests that value improvement efforts in geriatric surgical care could be particularly targeted at high-resource specialties.

We also observed a steady increase in the proportion of surgical admissions among elderly ICU patients over time, consistent with long-term trends reported in other aging societies [21,22]. This shift has important clinical and policy implications. Evidence from multiple settings suggests that a substantial proportion of elderly patients prioritize comfort and quality of life over maximal life extension, yet preferences are often not explicitly elicited before ICU admission [23]. In China and other Asian contexts, family-centered decision-making and a strong cultural impetus to “do everything possible” may further increase the risk of non-beneficial escalation, particularly in perioperative settings where the trajectory can change rapidly. Our results reinforce the importance of early, structured goals-of-care discussions and shared decision-making that incorporate prognosis, expected postoperative recovery, patient values, and the likely resource intensity of treatment. From a policy perspective, ongoing reforms such as diagnosis-related group (DRG) and diagnosis-intervention packet (DIP) payment models should explicitly consider the higher and more variable resource demands associated with geriatric surgery. Otherwise, uniform reimbursement may either undercompensate hospitals caring for high-complexity elderly surgical patients or unintentionally incentivize inefficient care patterns, thereby affecting equity in ICU access.

Although adjusted analyses suggested stable inflation-adjusted expenditures over time, unadjusted costs showed an annual increase of 2.84%, accompanied by a substantial shift in cost composition. Specifically, consumables increased from 32.0% of total costs in 2014 to 42.0% in 2021, while the share of drug expenditures declined from 36.9% to 22.1%. This structural shift likely reflects the combined effects of evolving clinical practice (greater uptake of technology-intensive interventions) and China’s sequential health reforms that altered pricing incentives and hospital revenue structures [24,25]. In particular, policies promoting medical–pharmaceutical separation (MPS) reduced the profit incentive associated with prescribing, plausibly contributing to the sustained decline in the proportion of drug expenditures. At the same time, higher utilization of devices and procedure-related materials—especially in an elderly population with rising surgical admissions—would be expected to increase consumable spending. Importantly, we observed an inflection around 2019, when Beijing implemented a zero-markup policy for medical consumables alongside mandated increases in fees for physician, surgical, and technical services. In our data, the rise in consumables slowed after 2019 while labor-related service fees increased, consistent with the intended policy direction of shifting expenditure away from materials and towards medical labor—a pattern also observed in other hospital-based studies in Beijing [26]. Taken together, our cost breakdown illustrates how macro-level policy reforms can translate into measurable changes in the microeconomics of ICU care, and why overall expenditure control may remain challenging even when specific cost categories are successfully constrained.

Despite high baseline mortality among elderly ICU patients, we documented a significant decline in hospital mortality from 19.5% in 2014 to 8.8% in 2021. This trend aligns with international reports of improving ICU survival rates in elderly patients [3,7,27], likely attributable to advances in perioperative care, sepsis management, organ support, and broader insurance coverage. A pivotal and related finding of our study is that this substantial improvement in patient outcomes was achieved without a concurrent increase in real, case-mix-adjusted healthcare expenditures. Our cost-trend analysis revealed a critical distinction: while nominal expenditures rose, real (inflation-adjusted) resource utilization remained stable over time. This indicates that the observed increase in monetary outlay was driven by general price inflation, not by an increase in the quantity or intensity of care provided. Consequently, the stability of real resource consumption alongside a marked mortality decline directly challenges the perception that intensive care for elderly patients is inherently cost-ineffective. However, as long-term functional outcomes and quality of life were not assessed, a comprehensive evaluation of ‘value’ would require integration of these patient-centered measures in future studies

Several factors were associated with higher costs among elderly patients. Consistent with prior evidence [28,29], the need for advanced organ support (notably mechanical ventilation) is a major cost driver and may help explain the higher expenditures observed in respiratory-related diagnoses. In contrast, cardiovascular diagnoses were linked to lower costs, likely due to the inclusion of post-operative cardiac and vascular surgery patients, whose ICU courses tend to be more stable and predictable. Among elderly patients, greater illness severity and comorbidity burden were associated with increased expenditures [2,13], likely reflecting more complex organ dysfunction and prolonged need for supportive therapy. Interestingly, older age within the elderly cohort was associated with lower costs, which may reflect earlier mortality, more conservative treatment trajectories, or both. The reasons why male sex predicted higher costs remain unclear and warrant further investigation.

This study has several limitations. First, its conduct within a single tertiary academic center in Beijing limits generalizability. ICU admission practices, technological capabilities, and, most critically, the structure of health insurance and hospital reimbursement—which directly drove the profound shifts in cost composition we observed—vary across regions in China and differ fundamentally from systems in other countries. Therefore, while the clinical phenomenon of surgery drastically altering cost profiles in the elderly is likely widely relevant, the exact cost magnitudes and proportions reported here are context-specific. Second, our economic analysis was confined to the index hospitalization and could not capture post-discharge costs, readmissions, or long-term functional outcomes. This is a significant omission, particularly for elderly ICU survivors who face elevated risks of post-intensive care syndrome (PICS), long-term care dependence, and associated costs [7,30,31,32]. Future studies incorporating long-term follow-up and societal perspectives are needed. Third, the retrospective design precluded the collection of data on patient and family preferences regarding life-sustaining treatments. As prior studies indicate that choices favoring comfort care are associated with significantly lower ICU costs and shorter stays [15]. The absence of this variable limits our ability to fully interpret cost variations and underscores the importance of integrating patient goals into decision-making for truly value-concordant care. Fourth, although baseline ADL was included, comprehensive frailty measures were unavailable, and residual confounding by unmeasured frailty cannot be excluded. Finally, from a methodological standpoint, we used the overall Beijing Consumer Price Index for inflation adjustment. Although sensitivity analyses confirmed the robustness of our primary conclusions, and the general CPI is a widely accepted proxy in health economics research in China [24,33]. The lack of a medical-service-specific index remains a limitation. Future studies employing more granular price deflators would allow for even more precise estimation of real cost trends. In addition, allocating all hospitalization costs to the year of first ICU admission is a simplification for admissions spanning two calendar years; however, sensitivity analyses excluding year-end spanning hospitalizations yielded consistent results.

Despite these limitations, our study provides robust severity-matched comparisons of healthcare costs across age groups in a large ICU cohort and offers longitudinal evidence on how expenditures and outcomes evolved during a period of major healthcare reform in China. These findings support patient-centered perioperative decision-making, inform reimbursement design under DRG/DIP reforms, and guide resource allocation strategies for aging ICU populations.

## 5. Conclusions

Our findings have important implications for clinical practice and health policy. The sharp cost escalation associated with surgery in elderly ICU patients underscores the need for careful preoperative evaluation and shared decision-making. Policymakers should account for the evolving cost composition of ICU care, particularly the increasing burden of consumables, when planning healthcare financing strategies. Finally, the decline in mortality highlights the potential for continued improvements in outcomes, reinforcing the value of investing in ICU care for the elderly, provided that treatment decisions remain patient-centered.

## Figures and Tables

**Table 1:** Matching of Elderly (≥80), Older (65–79) and Younger ICU patients (16–64).

Matching Criteria	16–64 Yr (*n* = 3398)	65–79 Yr (*n* = 3398)	≥80 Yr (*n* = 3398)	*p*
Male, *n* (%)	1919 (56.5)	1919 (56.5)	1919 (56.5)	_
Surgical patients, *n* (%)	1323 (38.9)	1323 (38.9)	1323 (38.9)	_
CCI, *n* (%)				_
Low (0)	346 (10.2)	346 (10.2)	346 (10.2)	
Medium (1–2)	1525 (44.9)	1525 (44.9)	1525 (44.9)	
High (≥3)	1527 (44.9)	1527 (44.9)	1527 (44.9)	
APS, median (IQR)	5.0 (3.0–8.0)	5.0 (3.0–8.0)	5.0 (3.0–8.0)	0.581

CCI, Charlson comorbidity index; APS, acute physiology score.

**Table 2:** Comparisons of Admission Characteristics, Intensive Care Unit (ICU) Stay Characteristics, and Outcomes: Elderly Patients (≥80) Versus Matched Older (65–79) and Younger (16–64) ICU Patients.

Characteristic	16–64 Yr (*n* = 3398)	65–79 Yr (*n* = 3398)	≥80 Yr (*n* = 3398)	*p*
Age, median (IQR)	56 (48–60)	71 (67–75)	83 (81–86)	<0.001
BMI, median (IQR)	24.9 (22.5–27.5) *^a^*	24.5 (22.0–26.9) *^b^*	23.6 (20.8–26.2) *^c^*	<0.001
ER admission, *n* (%)	2499 (73.5)	2445 (72.0)	2762 (81.3)	<0.001
Insured patients, *n* (%)	2448 (72.0) *^a^*	2508 (73.8) *^a^*	3908 (85.6) *^b^*	<0.001
ADL at admission, median(IQR)	60 (30–95)	55 (30–90)	45 (25–65)	<0.001
Principal diagnosis, *n* (%)				<0.001
Infectious	263 (7.7)	279 (8.2)	455 (13.4)	
Cardiovascular	1274 (37.5)	1320 (38.8)	1364 (40.1)	
Respiratory	114 (3.4)	166 (4.9)	257 (7.6)	
Gastrointestinal	231 (6.8)	220 (6.5)	374 (11.0)	
Neurologic	769 (22.6)	723 (21.3)	419 (12.3)	
Hemotologic and oncologic	576 (17.0)	539 (15.9)	299 (8.8)	
Musculoskeletal and injuries	61 (1.8)	90 (2.6)	163 (4.8)	
Other diseases	110 (3.2)	61 (1.8)	67 (2.0)	
Organ support, *n* (%)				
Invasive ventilation	748 (22.0) *^a^*	784 (23.1) *^a^*	644 (19.0) *^b^*	<0.001
RRT	85 (2.5)	62 (1.8)	60 (1.8)	0.042
Inotropics/vasopressors	557 (16.4) *^a^*	705 (20.7) *^b^*	765 (22.5) *^b^*	<0.001
Enteral nutrition support	792 (23.3)	827 (24.3)	763 (22.5)	0.099
Parenteral nutrition	867 (25.5) *^a^*	920 (27.1) *^a^*	1078 (31.7) *^b^*	<0.001
Tracheostomy	218 (6.4) *^a^*	165 (4.9) *^b^*	98 (2.9) *^c^*	<0.001
Blood transfusion, *n* (%)				
Red blood cells	338 (9.9) *^a^*	390 (11.5) *^a^*	497 (14.6) *^b^*	<0.001
Plasma	359 (10.6) *^a^*	382 (11.2) *^ab^*	423 (12.4) *^b^*	0.04
Platelets	95 (2.8)	94 (2.8)	73 (2.1)	0.151
Albumin Infusion	795 (23.4) *^a^*	953 (28.0) *^b^*	1044 (30.7) *^c^*	<0.001
ICU LOS, median (IQR), d	3.1 (1.6–7.4) *^a^*	3.8 (1.7–8.1) *^b^*	4.5 (2.0–9.0) *^c^*	<0.001
Hospital LOS, median (IQR), d	11.1 (7.7–16.8) *^a^*	11.9 (7.9–17.9) *^b^*	11.4 (7.5–17.0) *^a^*	0.004
ICU mortality, *n* (%)	159 (4.7) *^a^*	245 (7.2) *^b^*	391 (11.5) *^c^*	<0.001
Hospital mortality, *n* (%)	176 (5.2) *^a^*	282 (8.3) *^b^*	459 (13.5) *^c^*	<0.001

ER admission, Admission from emergency room; ADL, activities of daily living; RRT, renal replacement therapy; LOS, length of stay; Different superscript letters (*^a^*, *^b^*, *^c^*) in the same row indicate statistically significant pairwise differences (*p* < 0.05). Groups marked with shared letters (e.g., *^ab^*) show no statistical difference from groups labeled with either *^a^* or *^b^*. Non-normally distributed continuous variables were compared using Friedman tests, followed by Wilcoxon signed-rank tests with Bonferroni correction. Categorical variables were compared using Cochran’s Q tests, followed by McNemar tests with Bonferroni correction.

**Table 3:** Comparisons of Medical Expenses and Expense Items: Elderly Patients (≥80) Versus Matched Older (65–79) and Younger (16–64) ICU Patients.

	16–64 Yr	65–79 Yr	≥80 Yr	*p*
Overall cost per patient, CNY, mean (SD)				
Total cost	63,779 (62,872) *^a^*	65,905 (67,980) *^a^*	62,567 (69,088) *^b^*	<0.001
Cost/day	5412 (6687) *^a^*	5366 (8849) *^a^*	5272 (7044) *^b^*	<0.001
Cost/survivor	63,041 (60,304) *^a^*	64,431 (62,463) *^a^*	59,493 (61,145) *^b^*	<0.001
Cost/decedent	77,274 (98,000)	82,184 (111,018)	82,248 (104,736)	0.429
Cost/surgery	82,594 (68,814) *^a^*	87,236 (69,292) *^b^*	90,494 (76,450) *^b^*	0.003
Cost/medical	51,782 (55,553) *^a^*	52,304 (63,508) *^a^*	44,761 (57,251) *^b^*	<0.001
Proportional breakdown of medical expense (%)				
Drug	29.9	29.7	29.2	
Surgery	4.7	4.2	3.4	
Laboratory	9.2	9.5	10.2	
Imaging	2.6	2.6	2.3	
Consumable	39.3	39	38.6	
Bed, monitoring and oxygen	9.1	9.8	10.8	
Nursing	2.5	2.5	2.8	
Others	2.6	2.6	2.7	

CNY, Chinese Yuan; Others cost, diagnosis, registration and other treatment. Different superscript letters (*^a^*, *^b^*) in the same row denote statistically significant differences (*p* < 0.05). Comparisons of healthcare costs after logarithmic transformation among the three age groups were performed using repeated-measures analysis of variance (RM-ANOVA), followed by paired-samples *t*-tests with Bonferroni adjustment. Independent-samples *t*-tests were used only for comparisons of costs after logarithmic transformation per survivor and per decedent.

**Table 4:** Multivariate model for predictors of healthcare cost in elderly patients.

Variable	β *^a^* (95% CI)	*p*
Age (older)	−0.011 (−0.019–−0.003)	0.005
Sex (male)	0.117 (0.061–0.173)	<0.001
APS	0.031 (0.025–0.036)	<0.001
CCI classification	0.134 (0.089–0.178)	<0.001
Admission type (Surgical)	0.911 (0.845–0.976)	<0.001
Principal diagnosis		
Cardiovascular	1	
Respiratory	0.114 (0.003–0.226)	0.045
Gastrointestinal	0.191 (0.095–0.287)	<0.001
Hemotologic and oncologic	0.113 (0.007–0.220)	0.037
Infectious	0.287 (0.197–0.378)	<0.001
Neurologic	0.305 (0.212–0.398)	<0.001
Musculoskeletal and injuries	0.475 (0.334–0.610)	<0.001
Other diseases	−0.157 (−0.359–0.044)	0.126
ICU admission date (continuous-reported per year)	−0.001 (−0.014–0.011)	0.829

APS, acute physiology score; ICU, intensive care unit; CCI classification, The Charlson Comorbidity Index classification was as follows: low (CCI = 0), moderate (CCI = 1–2), and high (CCI ≥ 3). *^a^* β-coefficient: represents the mean difference in log-transformed cost. To interpret the percentage difference in cost, apply the transformation: Percentage Change = (exp(β) − 1) × 100%. For example, males compared with females (after accounting for other covariates) β = 0.117 corresponds to an 12.4% higher cost. R^2^ = 0.276.

## Data Availability

All data generated or analyzed during this study are included in this published article.

## Supplementary Materials

The following supporting information can be downloaded at: https://www.mdpi.com/article/10.3390/healthcare14030364/s1, Table S1: Standardized mean differences (SMDs) for all matching variables across the three matched groups; Table S2: Association between age groups and log-transformed total medical costs relative to the elderly group; Table S3: Pairwise comparisons of age groups on log-transformed total medical costs relative to the elderly group, stratified by surgical status; Table S4: Fit statistics and variance components for the linear mixed-effects model; Table S5: Comparison of mixed-effects model vs. ordinary least squares regression; Table S6: Multivariable Linear Regression on Full Unmatched Cohort (N = 31,535); Table S7: Comparison of standard errors and inference: ordinary least squares vs. robust standard errors; Table S8: Validation of regression estimates using nonparametric bootstrap (500 replications); Table S9: Sensitivity analysis excluding influential observations (N = 31,219); Table S10: Association between age groups and log-transformed total medical costs relative to the elderly group (adjusted for admission ADL); Table S11: Characteristics of elderly patients: matched vs. unmatched; Table S12: Mixed effects Model for Surgical Cost Differentials by Diagnosis; Table S13: Estimated surgical cost premium by diagnosis among patients aged ≥80 years; Table S14: Characteristics and Cost Structure of Surgical Patients, Stratified by Age Group; Table S15: Distribution of patients whose index hospitalization spanned a calendar year-end, by age group and admission type; Table S16: Sensitivity analysis: Multivariate model for predictors of healthcare cost in elderly patients, excluding those with hospitalizations spanning calendar years; Table S17: Sensitivity analysis: comparison of price adjustment approaches; Table S18: Nominal vs. real cost trends in elderly ICU patients; Table S19: Multivariable logistic regression model for hospital mortality in elderly patients; Table S20: Sensitivity analysis: Multivariable logistic regression model for in hospital mortality in elderly patients (≥80 years), adjusted for admission Activities of Daily Living (ADL) score.
