# Supplementary material for: Healthcare Costs and Mortality Trends of Elderly ICU Patients: Evidence from an Eight-Year Cohort Study in China
Source: Healthcare (Basel). 2026 Jan 31;14(3):364. doi: 10.3390/healthcare14030364 (PMC12896854; doi:10.3390/healthcare14030364)
Supplement: Supplementary file 1 [file healthcare-14-00364-s001.zip › healthcare-4000430-supplementary.pdf]

Table S1. Standardized mean differences (SMDs) for all matching variables across the three matched groups.

Variable	SMD (≥80yr vs. 16–64yr)	SMD (≥80yr vs. 65–79yr)	SMD (65–79yr vs. 16–64yr)
Demographics			
Male, %	0	0	0
Surgical admission, %	0	0	0
Comorbidity			
CCI: Low (0)	0	0	0
CCI: Medium (1–2)	0	0	0
CCI: High (≥3)	0	0	0
CCI score, mean	0.103	0.019	0.084
APS score, mean	0.014	0.009	0.005
Primary diagnosis, %			
Infectious	0.185	0.168	0.017
Cardiovascular	0.054	0.026	0.028
Respiratory	0.186	0.111	0.077
Gastrointestinal	0.148	0.161	0.013
Neurologic	0.274	0.241	0.033
Hematologic and oncologic	0.245	0.216	0.029
Musculoskeletal and injuries	0.169	0.114	0.058
Other diseases	0.08	0.013	0.092

Note: The matching procedure successfully balanced the four prespecified matching variables (all SMDs <0.1). Greater residual heterogeneity was observed in some secondary characteristics (e.g., primary diagnosis), which were therefore adjusted for in all subsequent multivariate models.

Table S2. Association between age groups and log-transformed total medical costs relative to the elderly group.

Variable	β[a](95% CI)	P value
Fixed Effects		
Age group		
≥80 years	1(Ref)	
16–64 years	0.112 (0.061, 0.163)	<0.001
65–79 years	0.116 (0.066, 0.167)	<0.001
Surgical status		
No	1 (Ref)	
Yes	0.808 (0.747, 0.869)	<0.001
Interaction: Age group × Surgery		
16–64 years × Yes	-0.186 (-0.268, -0.105)	<0.001
65–79 years × Yes	-0.126 (-0.207, -0.045)	0.002
Covariates		
Principal diagnosis		
Cardiovascular	1 (Ref)	
Respiratory	-0.032 (-0.109, 0.045)	0.41
Gastrointestinal	0.003 (-0.061, 0.066)	0.933
Hematologic and oncologic	0.136 (0.080, 0.192)	<0.001
Infectious	0.276 (0.216, 0.335)	<0.001
Neurologic	0.435 (0.388, 0.481)	<0.001
Musculoskeletal and injuries	0.498 (0.399, 0.596)	<0.001
Other diseases	-0.016 (-0.126, 0.094)	0.773
Random Effects	Variance	
Intercept (Pair ID)	0.04	
Residual	0.67	

Abbreviation: CI, confidence interval.

Note: Results are from a linear mixed-effects model adjusted for all variables listed. The dependent variable was ln(cost). The elderly group (≥80 years) served as the reference category for age group comparisons. The model included a random intercept for matched pairs. [a]β-coefficient: represents adjusted mean difference in log-transformed cost. To interpret the percentage difference in cost, apply the transformation: Percentage Change = [exp(β) – 1] × 100%. For example, for surgical patients in the elderly group compared with non-surgical (after accounting for other covariates), β = 0.808 corresponds to a 124% higher cost.

Table S3. Pairwise comparisons of age groups on log-transformed total medical costs relative to the elderly group, stratified by surgical status.

Comparison	Surgical Status	Mean Difference (95% CI)	P value
16–64 years vs. ≥80 years	No	0.112 (0.061, 0.163)	<0.001
16–64 years vs. ≥80 years	Yes	-0.074 (-0.138, -0.010)	0.023
65–79 years vs. ≥80 years	No	0.116 (0.066, 0.167)	<0.001
65–79 years vs. ≥80 years	Yes	-0.010 (-0.073, 0.054)	0.762

Abbreviation: CI, confidence interval.

Note: Mean difference represents the estimate in log-transformed cost. Negative values indicate lower costs compared to the elderly group (≥80 years). P-values are derived from simple effects analysis following a significant age group × surgery interaction (P for interaction < 0.05).

Table S4. Fit statistics and variance components for the linear mixed-effects model.

Component	Estimate (95% CI)	Interpretation
Model Fit Statistics		
Marginal R^2	0.195	Fixed effects explain 19.5% of variance
Conditional R^2	0.28	Full model explains 28.0% of variance
Variance explained by matching	0.085	Additional 8.5% of variance explained by pair structure
Likelihood ratio test	$\chi^2(1) = 26.73$, <0.001	Random effects significantly improve model fit
Variance Components		
Between-pairs variance (τ^2)	0.037 (0.025, 0.056)	Variance attributable to differences between matched pairs
Within-pair variance (σ^2)	0.674 (0.651, 0.697)	Residual variance within matched pairs
Total variance	0.711	$\tau^2 + \sigma^2$ = total unexplained variance
Correlation Structure		
Intraclass correlation (ICC)	5.26%	Proportion of total variance between pairs: $\tau^2/(\tau^2 + \sigma^2)$
Within-pair correlation	0.053	Correlation between patients in the same matched pair

Abbreviation: CI, confidence interval.

Note: Results from a linear mixed-effects model with random intercepts for matched pairs. Marginal R^2 represents variance explained by fixed effects only; Conditional R^2 represents variance explained by both fixed and random effects. Variance components estimated using restricted maximum likelihood (REML). N = 10,194 patients nested in 3,398 matched pairs.

Table S5. Comparison of mixed-effects model vs. ordinary least squares regression.

Parameter	Mixed Model (REML)	OLS Model	Absolute Difference	Relative Difference (%)
Fixed Effects				
16–64 years vs. ≥80 years	0.112	0.111***	0.002	1.7
65–79 years vs. ≥80 years	0.116	0.115***	0.001	1.1
Surgical admission	0.808	0.807***	0.001	0.1
Interaction: Age group × Surgery				
16–64 years × Yes	-0.186	-0.186***	0	0
65–79 years × Yes	-0.126	-0.127**	0.001	0.4
Model Fit				
AIC	25,514	25,467	-46.96	—
BIC	25,622	25,561	-61.41	—
R^2 / Conditional R^2	0.28	0.195	—	—
ICC	5.26%	—	—	—
Likelihood ratio test	$\chi^2(1)$ = 26.73***	—	—	—

Note: **P < 0.001, *P < 0.01. The mixed model includes random intercepts for matched pairs. The OLS model ignores the pair structure. Differences calculated as Mixed – OLS. Percentage differences for coefficients calculated as [(Mixed – OLS) / OLS] × 100%. Conditional R^2 includes variance explained by both fixed and random effects.

Table S6. Multivariable Linear Regression on Full Unmatched Cohort (N = 31,535)

Variable	β (95% CI)	Robust SE	P	[a]Effect Sizea
Age Group				
≥80 years	1			
16–64 years	0.130 (0.084, 0.177)	0.023	<0.001	+13.9% (8.8, 19.4)
65–79 years	0.127 (0.080, 0.174)	0.024	<0.001	+13.5% (8.3, 19.0)
Surgical Admission	0.900 (0.846, 0.955)	0.028	<0.001	+146% (133, 160)
Interaction: Age Group × Surgery				
16–64 years ×Yes	-0.166 (-0.227, -0.104)	0.031	<0.001	-15.3%(-20.3, -9.9)
65–79 years ×Yes	-0.093 (-0.156, -0.030)	0.032	0.004	-8.9%(-14.5, -3.0)
Covariates				
Sex(male)	0.071 (0.054, 0.089)	0.009	<0.001	7.40%
CCI (per point)	0.044 (0.039, 0.049)	0.003	<0.001	+4.5% per point
APS (per point)	0.031 (0.029, 0.033)	0.001	<0.001	+3.1% per point
Primary Diagnosis				
Cardiovascular	1			
Infectious	-0.149 (-0.196, -0.101)	0.024	<0.001	-13.80%
Respiratory	0.003 (-0.037, 0.044)	0.021	0.868	0.30%
Gastrointestinal	0.024 (0.002, 0.047)	0.012	0.035	2.50%
Neurologic	0.111 (0.065, 0.156)	0.023	<0.001	11.70%
Hematologic/Oncologic	0.205 (0.180, 0.230)	0.014	<0.001	22.70%
Musculoskeletal/Injury	0.268 (0.215, 0.321)	0.031	<0.001	30.70%
Other	-0.223 (-0.288, -0.158)	0.033	<0.001	-20.00%
Constant	9.862 (9.819, 9.905)	0.022	<0.001	

CI: confidence interval; APS: acute physiology score; CCI: Charlson Comorbidity Index

Note: Model: ln(Total Cost) ~ Age Group + Surgical Status + Age×Surgery + Sex + CCI + APS + Primary Diagnosis. Inference: Coefficients (β) represent mean differences in log-transformed cost. [a]Effect Size: Percentage Change = [exp(β) − 1] × 100%. For example, β = 0.130 corresponds to a 13.9% higher cost. Robust (Huber–White) standard errors reported to account for heteroskedasticity (Breusch–Pagan test: $\chi^2 = 612.31$, $P < 0.001$). Model Fit: $R^2 = 0.240$; Adjusted $R^2 = 0.240$; $F(15, 31519) = 663.69$, $P < 0.001$.

Table S7. Comparison of standard errors and inference: ordinary least squares vs. robust standard errors.

Variable	β	OLS SE (95% CI)	Robust SE (95% CI)	P (OLS)	P (Robust)
Age Group					
≥80 years	1(Ref)	—	—	—	—
16–64 years	0.13	0.020 (0.091, 0.169)	0.023 (0.085, 0.175)	<0.001	<0.001
65–79 years	0.127	0.021 (0.087, 0.167)	0.024 (0.080, 0.174)	<0.001	<0.001
Surgical Admission	0.9	0.027 (0.847, 0.953)	0.028 (0.846, 0.955)	<0.001	<0.001
Interaction: Age Group × Surgery					
16–64 years × Yes	-0.166	0.030 (-0.224, -0.108)	0.031 (-0.227, -0.104)	<0.001	<0.001
65–79 years × Yes	-0.093	0.031 (-0.154, -0.032)	0.032 (-0.156, -0.030)	0.003	0.004
Covariates					
Sex (Male)	0.071	0.009 (0.054, 0.089)	0.009 (0.054, 0.089)	<0.001	<0.001
CCI (per point)	0.044	0.003 (0.039, 0.049)	0.003 (0.039, 0.049)	<0.001	<0.001
APS (per point)	0.031	0.001 (0.029, 0.033)	0.001 (0.029, 0.033)	<0.001	<0.001
Primary Diagnosis					
Cardiovascular	1(Ref)	—	—	—	—
Infectious	-0.149	0.026 (-0.199, -0.098)	0.024 (-0.196, -0.101)	<0.001	<0.001
Respiratory	0.003	0.020 (-0.037, 0.044)	0.021 (-0.037, 0.044)	0.868	0.868
Gastrointestinal	0.024	0.013 (0.002, 0.047)	0.012 (0.002, 0.047)	0.035	0.035
Neurologic	0.111	0.020 (0.065, 0.156)	0.023 (0.065, 0.156)	<0.001	<0.001
Hematologic/Oncologic	0.205	0.013 (0.180, 0.230)	0.014 (0.180, 0.230)	<0.001	<0.001
Musculoskeletal/Injury	0.268	0.027 (0.215, 0.321)	0.031 (0.215, 0.321)	<0.001	<0.001
Other	-0.223	0.030 (-0.288, -0.158)	0.033 (-0.288, -0.158)	<0.001	<0.001

Note: This table compares inference for key variables from the full-sample model (see Table S6) under homoscedastic (OLS) and heteroscedasticity-robust assumptions. Robust SEs are generally larger, yet all key findings remain highly significant, demonstrating robustness to heteroscedasticity.

Table S8. Validation of regression estimates using nonparametric bootstrap (500 replications).

Variable	β Coefficient (95% CI)	Bootstrap SE	Z Value	P value
Age Group				
≥80 years	1 (Ref)	—	—	—
16–64 years	0.130 (0.083, 0.177)	0.024	5.38	<0.001
65–79 years	0.127 (0.080, 0.175)	0.024	5.26	<0.001
Surgical Admission	0.900 (0.849, 0.951)	0.026	34.74	<0.001
Interaction: Age Group × Surgery				
16–64 years × Yes	-0.166 (-0.227, -0.105)	0.031	-5.3	<0.001
65–79 years × Yes	-0.093 (-0.155, -0.031)	0.032	-2.92	0.004
Covariates				
Sex (Male)	0.071 (0.054, 0.088)	0.009	8.21	<0.001
CCI (per point)	0.044 (0.039, 0.049)	0.003	17.41	<0.001
APS (per point)	0.031 (0.029, 0.033)	0.001	31.62	<0.001
Primary Diagnosis				
Cardiovascular	1 (Ref)	—	—	—
Infectious	-0.149 (-0.197, -0.100)	0.025	-6.01	<0.001
Respiratory	0.003 (-0.037, 0.043)	0.02	0.17	0.866
Gastrointestinal	0.024 (0.002, 0.047)	0.012	2.09	0.036
Neurologic	0.111 (0.067, 0.155)	0.023	4.89	<0.001
Hematologic/Oncologic	0.205 (0.177, 0.232)	0.014	14.68	<0.001
Musculoskeletal/Injury	0.268 (0.207, 0.328)	0.031	8.67	<0.001
Other	-0.223 (-0.289, -0.157)	0.034	-6.64	<0.001
Constant	9.862 (9.819, 9.905)	0.022	446.41	<0.001

Abbreviations: APS, acute physiology score; CCI: Charlson Comorbidity Index.

Note: Estimates derived from nonparametric bootstrap resampling (500 replications). Bootstrap standard errors (SEs) and 95% confidence intervals (CIs) provide inference free of parametric assumptions about error distribution. Results are essentially identical to the robust regression estimates in Table S6, providing strong independent validation of the primary findings.

Table S9. Sensitivity analysis excluding influential observations (N = 31,219).

Variable	β Coefficient (95% CI)	Robust SE	P value
Age Group			
≥80 years	1(Ref)	—	—
16–64 years	0.142 (0.105, 0.179)	0.019	<0.001
65–79 years	0.124 (0.085, 0.162)	0.02	<0.001
Surgical Admission	0.893 (0.843, 0.943)	0.026	<0.001
Interaction: Age Group × Surgery			
16–64 years × Yes	-0.190 (-0.245, -0.135)	0.028	<0.001
65–79 years × Yes	-0.099 (-0.157, -0.041)	0.03	0.001
Covariates			
Sex (Male)	0.074 (0.057, 0.090)	0.008	<0.001
CCI (per point)	0.043 (0.038, 0.048)	0.002	<0.001
APS Score (per point)	0.033 (0.032, 0.035)	0.001	<0.001
Primary Diagnosis			
Cardiovascular	1(Ref)	—	—
Infectious	-0.166 (-0.214, -0.118)	0.025	<0.001
Respiratory	-0.021 (-0.057, 0.016)	0.019	0.26
Gastrointestinal	0.018 (-0.006, 0.042)	0.012	0.144
Neurologic	0.078 (0.041, 0.115)	0.019	<0.001
Hematologic/Oncologic	0.196 (0.172, 0.220)	0.012	<0.001
Musculoskeletal/Injury	0.337 (0.286, 0.389)	0.026	<0.001
Other Diseases	-0.262 (-0.319, -0.205)	0.029	<0.001
Constant	9.873 (9.836, 9.911)	0.019	<0.001

Abbreviations: APS, acute physiology score; CCI: Charlson Comorbidity Index.

Note: This sensitivity analysis excludes 316 observations (1.0% of full sample) identified as influential (Cook's D > 4/N). Results using robust standard errors demonstrate that all key findings regarding age group effects, surgical costs, and their interaction remain unchanged in direction and statistical significance, confirming robustness to potential outliers.

Table S10. Association between age groups and log-transformed total medical costs relative to the elderly group (adjusted for admission ADL).

Variable	β (95% CI)	P value
Fixed Effects		
Age group		
≥80 years	1 (Reference)	—
16–64 years	0.169 (0.118, 0.221)	<0.001
65–79 years	0.145 (0.095, 0.196)	<0.001
Surgical status		
No	1 (Reference)	—
Yes	0.883 (0.822, 0.945)	<0.001
Interaction: Age group × Surgery		
16–64 years × Yes	–0.171 (–0.253, –0.090)	<0.001
65–79 years × Yes	–0.087 (–0.168, –0.006)	0.035
Principal diagnosis		
Cardiovascular	1 (Reference)	—
Respiratory	–0.026 (–0.103, 0.051)	0.506
Gastrointestinal	0.006 (–0.057, 0.069)	0.855
Hematologic and oncologic	0.205 (0.148, 0.262)	<0.001
Infectious	0.234 (0.175, 0.294)	<0.001
Neurologic	0.318 (0.268, 0.367)	<0.001
Musculoskeletal and injuries	0.431 (0.333, 0.529)	<0.001
Other diseases	–0.048 (–0.158, 0.061)	0.385
Admission ADL (per point)	–0.0042 (–0.0048, –0.0036)	<0.001
Random Effects	Variance	
Intercept (Pair ID)	0.025	
Residual	0.673	

Abbreviation: CI, confidence interval; ADL, Activities of Daily Living.

Note: Results are from a linear mixed-effects model adjusted for all variables listed, including admission ADL score. The dependent variable was ln(cost). The elderly group (≥80 years) served as the reference category for age group comparisons. The model included a random intercept for matched pairs. β-coefficient represents adjusted mean difference in log-transformed cost. To interpret the percentage difference in cost, apply the transformation: Percentage Change = [exp(β) – 1] × 100%. For example, for surgical patients in the elderly group compared with non-surgical patients (after accounting for other covariates), β = 0.883 corresponds to a 142% higher cost.

Table S11. Characteristics of elderly patients: matched vs. unmatched.

Variable	Unmatched Elderly (n = 226)	Matched Elderly (n = 3,398)	P value
Demographics			
Age, median (IQR)	83 (81–86)	83 (81–86)	0.433
Male, n (%)	2 (0.9)	1,920 (56.5)	<0.001
Surgical admission, n (%)	1 (0.4)	1,323 (38.9)	<0.001
CCI, n (%)			
Low (0)	3 (1.3)	346 (10.2)	<0.001
Medium (1–2)	3 (1.3)	1,525 (44.9)	<0.001
High (≥3)	220 (97.3)	1,527 (44.9)	<0.001
CCI score, median (IQR)	4 (3–5)	2 (1–4)	<0.001
Severity			
APACHE II score, median (IQR)	17 (14–20)	13 (10–18)	<0.001
APS score, median (IQR)	6 (3–9)	5 (3–8)	<0.001
Primary diagnosis, n (%)			<0.001
Infectious	39 (17.3)	455 (13.4)	
Cardiovascular	102 (45.1)	1,364 (40.1)	
Respiratory	26 (11.5)	257 (7.6)	
Gastrointestinal	11 (4.9)	374 (11.0)	
Neurologic	31 (13.7)	419 (12.3)	
Hematologic and oncologic	12 (5.3)	299 (8.8)	
Musculoskeletal and injuries	2 (0.9)	163 (4.8)	
Other diseases	3 (1.3)	67 (2.0)	
Organ support, n (%)			
Invasive ventilation	40 (17.7)	644 (19.0)	0.641
RRT	6 (2.7)	60 (1.8)	0.333
Inotropics/vasopressors	54 (23.9)	765 (22.5)	0.631
Outcomes & Costs			
ICU LOS, median (IQR), d	6.2 (3.5–11.3)	4.5 (2.0–9.0)	<0.001
Hospital LOS, median (IQR), d	11.9 (8.0–16.0)	11.5 (7.5–17.1)	0.895
ICU mortality, n (%)	44 (19.5)	391 (11.5)	<0.001
Hospital mortality, n (%)	52 (23.0)	459 (13.5)	<0.001
Total cost, CNY, mean (SD)	40,392 (37,542)	62,567 (69,088)	<0.001
Total cost, CNY, median (IQR)	28,387 (16,889–51,040)	42,118 (21,049–75,643)	<0.001

Abbreviations: APS, acute physiology score; CCI, Charlson Comorbidity Index; CNY, Chinese Yuan; IQR, interquartile range; LOS, length of stay; RRT, renal replacement therapy.

Note: Unmatched patients were those who could not be matched 1:1:1 based on sex, surgical status, CCI category, and APS (±1). The unmatched group had higher severity, lower surgical rates, and lower costs, indicating that their exclusion likely created a conservative bias for the primary cost comparison.

Table S12. Mixed-effects Model for Surgical Cost Differentials by Diagnosis

Variable	β (95% CI)	P value	Cost increase
Fixed Effects			
Age group (ref: ≥80 years)			
16–64 years	0.043 (0.003, 0.083)	0.036	4.40%
65–79 years	0.069 (0.030, 0.109)	0.001	7.20%
Surgical status (ref: No)	0.932 (0.875, 0.989)	<0.001	154.00%
Principal diagnosis (ref: Cardiovascular)			
Gastrointestinal	0.096 (0.021, 0.171)	0.012	10.10%
Neurologic	0.599 (0.538, 0.659)	<0.001	82.00%
Musculoskeletal/injuries	0.701 (0.525, 0.878)	<0.001	101.60%
Hematologic/oncologic	0.237 (0.118, 0.356)	<0.001	26.70%
Infectious	0.378 (0.315, 0.441)	<0.001	45.90%
Respiratory	0.036 (−0.044, 0.116)	0.379	3.70%
Other diseases	0.214 (0.084, 0.344)	0.001	23.90%
Interaction: Surgery × Diagnosis			
Gastrointestinal × Yes	−0.296 (−0.434, −0.158)	<0.001	−25.6%
Neurologic × Yes	−0.425 (−0.520, −0.331)	<0.001	−34.6%
Musculoskeletal/injuries × Yes	−0.389 (−0.602, −0.176)	<0.001	−32.2%
Hematologic/oncologic × Yes	−0.278 (−0.414, −0.142)	<0.001	−24.3%
Infectious × Yes	−0.698 (−0.894, −0.503)	<0.001	−50.2%
Respiratory × Yes	−0.411 (−0.700, −0.121)	0.005	−33.7%
Other diseases × Yes	−0.777 (−1.018, −0.536)	<0.001	−54.0%
Random Effects	Variance		
Intercept (Pair ID)	0.035		
Residual	0.668		

Table S13. Estimated surgical cost premium by diagnosis among patients aged ≥80 years.

Principal diagnosis	β (95% CI)	Percentage increase	P value
Cardiovascular	0.932 (0.875, 0.989)	154.00%	<0.001
Gastrointestinal	0.636 (0.566, 0.706)	89.00%	<0.001
Hematologic/oncologic	0.654 (0.584, 0.724)	92.30%	<0.001
Neurologic	0.507 (0.437, 0.577)	66.10%	<0.001
Musculoskeletal/injuries	0.543 (0.472, 0.614)	72.10%	<0.001
Infectious	0.234 (0.164, 0.304)	26.40%	<0.001
Respiratory	0.521 (0.451, 0.591)	68.40%	<0.001
Other diseases	0.155 (0.085, 0.225)	16.80%	<0.001

Notes: The dependent variable was ln(total cost). Percentage change was calculated as [exp(β) − 1] × 100%. In Table S12, the main effect for surgical status represents the surgical vs. non-surgical difference within the reference diagnosis category (cardiovascular). For other diagnoses, the total surgical effect equals the main surgical coefficient plus the corresponding interaction term. Table S13 reports the total surgical effect (β) for each diagnosis within the ≥80 years group. Interpret estimates for respiratory surgery (n = 3) and other diseases surgery (n = 19) with caution due to limited sample size. All P values are two-sided.

Table S14. Characteristics and Cost Structure of Surgical Patients, Stratified by Age Group

Characteristic	16–64Yr (n=1,323)	65–79Yr (n=1,323)	≥80Yr (n=1,323)	P value
Age, median (IQR)	56 (49–61)	71 (67–75)	83 (81–86)	<0.001
BMI, median (IQR)	24.6 (22.3–27.1)	24.4 (22.0–26.6)	23.6 (21.0–26.0)	<0.001
Insured patients, n (%)	961 (72.6)	971 (73.4)	1129 (85.3)	<0.001
ADL, median (IQR)	95 (50–100)	90 (50–100)	55 (30–85)	<0.001
Principal Diagnosis, n (%)				<0.001
Cardiovascular	361 (27.3)	382 (28.9)	501 (37.9)	
Gastrointestinal	45 (3.4)	56 (4.2)	138 (10.4)	
Musculoskeletal and injuries	33 (2.5)	59 (4.5)	133 (10.1)	
Neurologic	288 (21.8)	325 (24.6)	251 (19.0)	
Hematologic and oncologic	503 (38.0)	460 (34.8)	245 (18.5)	
Infectious	35 (2.6)	16 (1.2)	33 (2.5)	
Respiratory	25 (1.9)	8 (0.6)	3 (0.2)	
Other diseases	33 (2.5)	17 (1.3)	19 (1.4)	
Organ support, n (%)				
Invasive ventilation	344 (26.0)[a]	353 (26.7)[a]	300 (22.7)[b]	<0.001
RRT	17(1.3)	17(1.3)	16(1.2)	0.98
Inotropics/vasopressors	216 (16.3)[a]	298 (22.5)[b]	309 (23.4)[b]	<0.001
Tracheostomy	56(4.2)[a]	53(4.0)[a]	37(2.8)[b]	0.108
Blood transfusion, n (%)	246 (18.6)[a]	285 (21.5)[b]	341 (25.8)[c]	<0.001
ICU LOS, median (IQR), d	1.9(0.9–4.7)[a]	1.9(0.9–4.7)[a]	2.8(1.0–5.6)[b]	<0.001
Hospital LOS, median (IQR), d	12.8(8.1–19.0)[a]	13.5(8.9–20.0)[a]	11.8(7.2–18.7)[b]	0.004
ICU mortality, n (%)	27 (2.0)[a]	51 (3.9)[b]	86 (6.5)[c]	<0.001
Hospital mortality, n (%)	33 (2.5)[a]	61 (4.6)[b]	115 (8.7)[c]	<0.001
Cost Analysis				
Total cost per patient, CNY, mean (SD)	82,594 (68,814)[a]	87,236 (69,292)[b]	90,494 (76,450)[b]	0.003
Cost Structure (%)				
Consumables	49.9	52.1	56	
Drugs	24	23.1	19.9	
Laboratory	6.6	6.4	6.5	
Surgical fee	7.3	6.4	5.4	
Bed, monitoring & oxygen	6	6	6.3	
Imaging	2.2	2.2	1.8	
Nursing	1.7	1.7	1.9	
Others	2.4	2.2	2.2	

Abbreviations: ADL, activities of daily living; BMI, body mass index; CNY, Chinese Yuan; ICU, intensive care unit; IQR, interquartile range; LOS, length of stay; SD, standard deviation.

Notes: [a, b, c]Different superscript letters within a row denote statistically significant pairwise differences ($P < 0.05$) based on post-hoc tests with Bonferroni adjustment. Groups marked with shared letters (e.g., *ab*) show no statistical difference from groups labeled with either *a* or *b*.

Statistical tests: Given the 1:1:1 matched design of the parent cohort, non-normally distributed continuous variables were analyzed using the Friedman test, with the Wilcoxon signed-rank test and Bonferroni adjustment for post-hoc comparisons. Categorical variables were compared using Cochran's Q test, with the McNemar test and Bonferroni adjustment for post-hoc pairwise comparisons.

Table S15. Distribution of patients whose index hospitalization spanned a calendar year-end, by age group and admission type.

Variable	16–64 years	65–79 years	≥80 years	Total
Total patients, n	3,398	3,398	3,398	10,194
Spanning year-end hospitalizations	99 (2.9%)	149 (4.4%)	144 (4.2%)	392 (3.8%)
admission type, n				
Surgical	41	54	64	159
Medical	58	95	80	233

Table S16. Sensitivity analysis: Multivariate model for predictors of healthcare cost in elderly patients, excluding those with hospitalizations spanning calendar years.

Variable	[a]β (95% CI)	P value
Age (per year)	–0.012 (–0.020 to –0.004)	0.003
Sex (male vs. female)	0.110 (0.053 to 0.167)	<0.001
APS (per point)	0.029 (0.023 to 0.035)	<0.001
Admission type (Surgical vs. Medical)	0.898 (0.831 to 0.965)	<0.001
Charlson Comorbidity Index (per category)	0.138 (0.092 to 0.183)	<0.001
Principal diagnosis (ref = Cardiovascular)		
Respiratory	0.115 (0.002 to 0.229)	0.047
Gastrointestinal	0.196 (0.099 to 0.294)	<0.001
Hematologic and oncologic	0.106 (–0.003 to 0.215)	0.057
Infectious	0.263 (0.170 to 0.355)	<0.001
Neurologic	0.298 (0.204 to 0.392)	<0.001
Musculoskeletal and injuries	0.496 (0.354 to 0.638)	<0.001
Other diseases	–0.174 (–0.376 to 0.029)	0.093
ICU admission year (per year)	–0.001 (–0.014 to 0.011)	0.833

APS, acute physiology score; ICU, intensive care unit; CCI classification, low (CCI = 0), moderate (CCI = 1–2), and high (CCI ≥3). [a]β-coefficient: represents the mean difference in log-transformed cost. To interpret the percentage difference in cost, apply the transformation: Percentage Change = (exp(β)－1)×100%. R2 =0.272.

Table S17. Sensitivity analysis: comparison of price adjustment approaches.

Parameter	CPI-Adjusted Costs (Primary)	Unadjusted Costs (Sensitivity)	Absolute Difference	Relative Difference (%)
Fixed Effects				
Age group (ref: ≥80 years)				
16–64 years	0.112 (0.061, 0.163)	0.105 (0.054, 0.156)	-0.007	-6.3
65–79 years	0.116 (0.066, 0.167)	0.116 (0.066, 0.167)	0	-0.2
Surgical admission	0.808 (0.747, 0.869)	0.818 (0.757, 0.879)	0.01	1.2
Interaction effects				
16–64 years × Yes	-0.186 (-0.268, -0.105)	-0.194 (-0.275, -0.113)	-0.008	4.3
65–79 years × Yes	-0.126 (-0.207, -0.045)	-0.144 (-0.224, -0.063)	-0.018	14.3
Model Fit				
Wald χ^2 (df = 12)	2,258.94	2,259.91	0.97	0.04
P value	<0.001	<0.001	—	—
Log restricted-likelihood	-12,742.00	-12,743.40	-1.45	—
Variance Components				
Between-pairs variance (τ^2)	0.037 (0.025, 0.056)	0.039 (0.027, 0.057)	0.002	5.1
Residual variance (σ^2)	0.674 (0.651, 0.697)	0.672 (0.650, 0.695)	-0.002	-0.2
Intraclass correlation (ICC)	5.26%	5.52%	0.26%	4.9
Likelihood ratio test	$\chi^2(1)$ = 26.73, <0.001	$\chi^2(1)$ = 29.43, <0.001	2.7	10.1

Abbreviation: CPI, Consumer Price Index.

Note: All coefficients are on the log scale. CPI-adjusted costs use the Beijing overall Consumer Price Index; unadjusted costs use nominal values. All key conclusions remain unchanged: surgical admission significantly increases costs (P < 0.001 in both analyses), and the magnitude of effect differs by only 1.2%.

Table S18. Nominal vs. real cost trends in elderly ICU patients.

Parameter	Unadjusted (Nominal)	CPI-Adjusted (Real)	Difference
Year coefficient, β (95% CI)	0.018 (0.005, 0.030)	-0.001 (-0.014, 0.011)	0.019
P value	0.006	0.829	—
Annual change, % (95% CI)	+1.8 (+0.5, +3.0)	-0.1 (-1.4, +1.1)	1.9
Interpretation	Significant increase	No significant trend	Inflation effect

Abbreviation: CPI, Consumer Price Index; CI, confidence interval.

Note: Nominal increases align with average Beijing inflation (2.1% per year), indicating stable real resource use over the study period.

Table S19. Multivariable logistic regression model for hospital mortality in elderly patients.

Variable	Odds Ratio (95% CI)	P value
Age (per year increase)	1.063 (1.032, 1.095)	<0.001
Sex (Male)	1.018 (0.809, 1.281)	0.881
APS (per point)	1.210 (1.186, 1.235)	<0.001
CCI classification	1.925 (1.576, 2.352)	<0.001
Surgical Admission	0.832 (0.627, 1.105)	0.204
Principal diagnosis		0.005
Cardiovascular	1(Ref)	
Respiratory	0.784 (0.503, 1.223)	0.284
Gastrointestinal	0.979 (0.648, 1.480)	0.921
Hematologic and oncologic	0.796 (0.493, 1.285)	0.351
Infectious	1.182 (0.853, 1.638)	0.315
Neurologic	0.473 (0.317, 0.705)	<0.001
Musculoskeletal and injuries	0.950 (0.537, 1.678)	0.859
Other diseases	0.635 (0.281, 1.431)	0.273
ICU admission year (per year)	0.860 (0.816, 0.906)	<0.001

Abbreviations: APS, acute physiology score; CCI, Charlson Comorbidity Index.

Note: Logistic regression analysis for in-hospital mortality (dependent variable). Independent variables: age, sex, APS, CCI classification, admission type, principal diagnosis, and ICU admission year. The Charlson Comorbidity Index classification was: low (CCI = 0), moderate (CCI = 1–2), high (CCI ≥3). ICU admission year was entered as a continuous variable; the odds ratio represents the change in odds of death per one-year increment.

Table S20. Sensitivity analysis: Multivariable logistic regression model for in-hospital mortality in elderly patients (≥80 years), adjusted for admission Activities of Daily Living (ADL) score.

Variable	Odds Ratio (95% CI)	P value
Age (per year increase)	1.058 (1.027 – 1.090)	<0.001
Sex (Male)	1.022 (0.812 – 1.287)	0.852
APS (per point)	1.198 (1.173 – 1.224)	<0.001
Charlson Comorbidity Index (per category)	1.888 (1.544 – 2.308)	<0.001
Surgical Admission	0.908 (0.681 – 1.210)	0.508
Principal diagnosis		0.001
Cardiovascular	Ref.	—
Respiratory	0.745 (0.475 – 1.168)	0.2
Gastrointestinal	0.938 (0.620 – 1.419)	0.762
Hematologic and oncologic	0.947 (0.581 – 1.546)	0.828
Infectious	1.104 (0.794 – 1.536)	0.555
Neurologic	0.389 (0.256 – 0.592)	<0.001
Musculoskeletal and injuries	0.906 (0.513 – 1.601)	0.734
Other diseases	0.606 (0.269 – 1.363)	0.226
ICU admission year (per year)	0.851 (0.808 – 0.897)	<0.001
Admission ADL (per point)	0.992 (0.987 – 0.997)	0.002

Abbreviations: ADL, Activities of Daily Living; APS, Acute Physiology Score; CI, confidence interval; ICU, intensive care unit.

Note: Charlson Comorbidity Index classification was: low (CCI = 0), moderate (CCI = 1–2), high (CCI ≥3). ICU admission year was entered as a continuous variable; the odds ratio represents the change in odds of death per one-year increment. ADL score ranges from 0 (complete dependence) to 100 (complete independence).
